# Independence or Contumacy?

**Published:** 1918-06-29

**Authors:** 


### INDEPENDENCE OR CONTUMACY?

BEFORE us is the annual report, delayed a little, as is usual in these times, of a medical institution which, six or seven years ago, was in a prosperous condition. It had come through the trials incidental to the early existence of many things, animate or inanimate, and under a devoted staff combined efficiency with detailed economy in a way which always leads to success. About the time of the passing of the National Insurance Act opportunity occurred of amalgamation with, or perhaps not exactly that, but rather taking into junior partnership, a neighbouring executive authority engaged in the same line of special work. Although the existing number of beds—and the institution had been planned from the beginning with a view to extension—could have been nearly doubled, the original trustees of the property would have been left with their responsible and authoritative status untouched. Given moderate additional representation on the general and house committees, a large accession of strength could have been secured: funds devoted to sending patients, on inconvenient journeys, to scattered places in various parts of the country would have gone instead to swell the funds of one central institution and make its position a sure one, and its technical equipment of high grade. But its authorities refused; they would not co-operate, would not sell outright either—would, in short, do nothing but continue, as the saying goes, "on their own." The staff are naturally discouraged, they feel themselves in a backwater: there are changes, changes too in the devoted attention to economy and to the stitch in time. Then the authorities feel bound to make an extension—a little one it has to be—and that swallows up a good deal of their reserve. About this time war supervenes. In every direction, at every small item, stress comes on the finances. The flow of charity slackens, while salaries have to be largely raised and employees humoured, whether worth humouring or no. In fine, for the first time, these last two years, since its earliest days, the institution shows a balance on the wrong side and begins to live on capital. Like many extravagant or unwise people in private life who find themselves in the same pickle, its directors will blame the war. But they are wrong; the regrettable plight of their charge is due to their unwillingness to see (although reminded by the Local Government Board) that union is strength.

We hope that a similar story will not have to be told of those special hospitals which treat in a similar way the advice of the administrators of King Edward's Fund. One of their spokesmen complains of unfair treatment. Now some very remarkable statements about the war and all proceeding therefrom have been current during the last four years. For instance, an obscure lady, one of the self-constituted publicists common nowadays, lately averred that juvenile misbehaviour was due to the children being overfed. And as the modern publicist is nothing if not ignorant, so her arguments were not troubled with the fact that German and Austrian children are unusually misbehaved in war-time too. But this complaint of unfair treatment by the representatives of a large section of the charitable public seems little less unreasonable. Is it unfair treatment to antagonise wasteful division of forces, and to encourage those who work to the best advantage in their power? And if we look to the future and to the trend of events, what is foreshadowed in the practice of medicine among the general population is the coming of a central authority, the Ministry of Health—and if that is well constituted, the sooner the better. It does not seem likely that such a body will show a mistaken leniency to individual predilections that those who administer King Edward's Fund properly refuse, but, rather, that pressure in an opposite direction will be considerably strengthened—and rightly so, for the period post-war will be no favourable one for a hospital's recovery from financial decrepitude.

